# Formulation and Evaluation of a Novel Matrix-Type Orally Disintegrating Ibuprofen Tablet 

**Published:** 2011

**Authors:** Hoda Tayebi, Seyed Alireza Mortazavi

**Affiliations:** School of Pharmacy, Shaheed Beheshti University of Medical Sciences, Tehran, Iran.

**Keywords:** Ibuprofen, Orally disintegrating tablet, Buccal drug delivery, Poly vinyl pyrrolidone, Xylitol, Croscarmellose, Physicochemical control

## Abstract

Orally disintegrating tablets (ODTs) are capable of turning quickly into a liquid dosage form in contact with the saliva, thus possessing the advantages of both the solid dosage forms particularly stability and liquid dosage forms specially ease of swallowing and pre-gastric absorption of drug. The aim of this study was to prepare a novel matrix-type buccal fast disintegrating ibuprofen tablet formulation using special polymers, water soluble excipients, super-disintegrants and quickly soluble granules. For this purpose different tablet formulations of ibuprofen were prepared. The amount of ibuprofen in each formulation was 100 mg. Eight groups of formulation were prepared (A-H series), accounting for a total number of 45 formulations. Formulations prepared were examined in terms of different physicochemical tests including powder/granule flowability, appearance, thickness, uniformity of weight, hardness, friability and disintegration time. Results of formulation F_22a_ (in series F), was found to be acceptable, making it the chosen formulation for further studies. Then, by adding various flavorants and sweeteners to this formulation, complementary series of formulations, named G and H, were prepared. Following the comparison of their taste with each other through asking 10 volunteers, the most suitable formulation regarding the taste, being formulation F_22s_, was chosen as the ultimate formulation. This formulation had PVP, ibuprofen and croscarmellose as the intra-granular components and xylitol and saccharin as the extra-granular ingredients. Formulation F_22s_ was found to be acceptable in terms of physicochemical tests conducted, showing quick disintegration within the buccal cavity, appropriate hardness and rather low friability. Hence formulation F_22s_ was selected as the final formulation.

## Introduction

Today, there are different drug delivery systems in the global drug market, aiming to facilitate the process of drug delivery and greater patient compliance. For systemic delivery, the oral route has been the preferred route of administration (1-4). Among these dosage forms are the orally disintegrating tablets (ODTs). ODTs are capable of turning quickly into a liquid dosage form in contact with the saliva (5, 6), resulting in pre-gastric absorption of drug so that more rapid onset of action and greater bioavailability would be expected (7). A more rapid onset of action has a significant effect on different clinical manifestations such as insomnia, anxiety, pain, fever, inflammation, *etc. *(8, 9). Increasing the drug bioavailability, which occurs due to immunization from liver first pass effect, is very important (6, 7). The side effects will be reduced due to a reduction of drug dosage, resulting in metabolite rate reduction in comparison to the usual dosage forms (9).

The target communities in using ODTs are children, the elderly, hospitalized patients, bodily and mental cripples, those with mastication and deglutition problems, patients with resistant chronic nausea, patients under chemotherapy, psychotic patients who hide their tablets beneath their tongue and those persons or travelers who have no access to water (6, 10, 11). ODTs have also been recently used in animals (6). 

Ibuprofen, a non-steroidal anti-inflammatory drug, has extensive use in adults and children, in order to overcome pain, fever and inflammation (8, 12, 13). The use of ODTs could help to reduce the gastrointestinal side effects of ibuprofen, since the tablet is disintegrated within the mouth (13-15).

Mizumoto *et al*. proved that saccharides can be an appropriate material for ODTs since they divided into high and low compressibility categories. Their use make it possible to achieve sufficient hardness, while maintaining the fast disintegration time (16). In another study, Zade *et al*. concluded that ODT of Tizanidine HCl can be successfully prepared by both the superdisintegration method and sublimation method, in addition to taste masking with eudragit E 100. However, in this study super-disintegration method was found to be superior to that of sublimation method (11). In other study conducted by Suresh *et al*., salbutamol sulphate tablets were prepared by wet granulation process using sublimable components (camphor and ammonium bicarbonate). All the prepared tablets were found to disintegrate fast. Showing disintegration time of less than a minute (17). 

In recent years, several new advanced technologies have been introduced for the formulation of ODTs with very interesting features such as extremely low disintegration time, exceptional taste masking ability, pleasant mouth feel and sugar free tablets for diabetic patients (6, 18). The technologies utilized for fabrication of ODTs include lyophilization, moulding, direct compression, cotton candy process, spray drying, sublimation, mass extrusion, nanonization and quick dissolve film formation. These techniques are based on the principles of increasing porosity and/or addition of super-disintegrants and water soluble excipients to the tablets. Some of these technologies are Zydis, Lyoc, Orasolv, Durasolv, Wowtab, Flashdose, Flashtab and Oraquick (6, 10, 18, 19). ‘Zydis’ was the first commercially available ODT in the drug market. It is a unique freeze-dried tablet in which the active drug is incorporated within a water-soluble matrix, which is then transformed into blister pockets and freeze dried to remove water by sublimation. ‘Orasolv’ technology involves taste masking of active drug. An effervescent disintegrating agent is also used in these tablets. Conventional blenders and tableting equipments are used for preparation of these tablets. Furthermore, a lower compaction force is used for manufacturing these tablets, in order to obtain soft and quickly disintegrating tablets. ‘Durasolv’ technology is one of the most suitable technologies to prepare products requiring low amounts of active ingredients. This technology uses drug, fillers and a lubricant to prepare the tablet. ‘Wow’ means ‘without water’. In the ‘Wowtab’ technology the active ingredients may constitute up to 50% w/w of the tablet. In this technique, saccharides of both low and high mouldability are used to prepare the granules. ‘Flashdose’ technology uses the combination of both Shearform and Ceform technologies in order to mask the bitter taste of the drug. A sugar based matrix, called ‘Floss’ is used, which is made up of a combination of excipients (crystalline sugars) alone or in combination with drug. In the ‘Flashtab’ technology microgranules of the taste-masked active drug are used. These taste-masked micro-crystals of active drug, disintegrating agent, a swelling agent and other excipients like soluble diluents are compressed to form a multiparticulate tablet that disintegrates rapidly. ‘Nanocrystal technology’ can also be used in the formulation of ODTs, and helps to improve compound activity and final product characteristics. Decreasing the particle size increases the surface area, which leads to an increase in dissolution rate. This goal can be accomplished predictably and efficiently using the Nanocrystal technology. Nanocrystal particles are small particles of drug substance, typically less than 1000 nm in diameter, which are produced by milling the drug, resulting in ODTs (20).

The formulations prepared from these techniques differ from each other on the basis of the factors like mechanical strength of the final product, drug and dosage form stability, mouth feel, taste and the period of time taken by the formulation to disintegrate or dissolve in the saliva (6).

There are some disadvantages to these technologies. The facilities needed to prepare some of them is too expensive or relatively expensive. Also, some technologies utilize special facilities and unique methods that are not easily available and practical for all companies. The low mechanical strength of some formulations makes very fragile tablets; hence the companies should develop a special handling and packaging system for these cases.

The aim of this study was to develop a novel and simple method for producing an ibuprofen ODT formulation, which besides having the mentioned advantages, is cost-effective and the facilities needed for their preparation are available for all pharmaceutical companies. For this purpose, a matrix-type ODT system was produced using special polymers, water soluble excipients, super-disintegrants and quickly soluble granules.

Although, numerous technologies had been developed for the fabrication of these unique dosage forms in the last two decades, but so far, no standardized technique has been designed or mentioned in the literature for their preparation and evaluation (18).

## Experimental

*Materials*

Ibuprofen powder *was purchased from Biocause Co. (Japan). Aspartame, croscarmellose, *silicone dioxide, magnesium stearate, polyethylene glycol 1000, glucose, sucrose, saccharin, sodium hydroxide, phenol phetalein, xylitol, isomalt, peach and strawberry essence *were all purchased from Merck Co. (Germany). *Methanol (HPLC grad) was *obtained *from LabScan Co. (Ireland). Alcohol 96% was *supplied by the *Parsian Shiraz Co. (Iran). Polyvinyl pyrrolidone *was purchased from Sigma Co. (USA)*.

*Studies on ibuprofen powder*

In the first stage of this study, physicochemical characteristics of ibuprofen powder including organoleptic properties, flowability, compressibility, disintegration time of the compacted powder and powder purity were investigated in the standard way (12, 21). 

*Preparation of ibuprofen ODT formulations*

Different formulations using various ingredients were prepared in 8 series (A-H), accounting for a total number of 45 formulations. The amount of ibuprofen in each formulation was 100 mg (22). Most formulations consisted of intra-granular (quickly soluble granules) and extra-granular parts. Ibuprofen ODT formulations were prepared using quickly soluble granules. Quickly soluble granules can dissolve in water within a few seconds, thus causing dissolution of the tablet. In general, the technology for making a matrix-type ibuprofen ODT formulation involved the use of special polymers, water-soluble excipients, super-disintegrants and quickly soluble granules. These components were throughly mixed and subsequently compressed into tablets, using a single punch tablet press equipped with 9 and 14 mm flat punches. At the beginning of the study, the 14 mm flat punch was used. As the time elapsed, formulation progress resulted in the use of a decreased amount of ingredients and as a result the 9 mm flat punch was used. 

The first series of formulations (series A) contained polyethylene glycol 1000, glucose or sucrose as the intra-granular part of formulation and ibuprofen, croscarmellose, magnesium stearate and silicone dioxide as the extra-granular part. Quickly soluble granules of series A formulations included two parts. One of them was polyethylene glycol 1000, which is a material with low melting point and the other was glucose or sucrose, which is a water-soluble material. These granules were made as follows: first of all polyethylene glycol 1000 was melted on a heater, then glucose or sucrose was added at room temperature under the action of a four-propeller mixer set at 50 rpm. After the preparation of granules, they were passed through a mesh 16 screen and stored in a beaker sealed with Para-film until use (23). Quickly soluble granules were used alongside other formulation ingredients. In the next stage, ibuprofen as the active ingredient, croscarmellose as the super-disintegrant, silicone dioxide as the glidant and magnesium stearate as the lubricant were screened and weighted separately. Then, all the mentioned components including the quickly soluble granules except for the lubricant, were mixed by the geometrical dilution method and finally lubricant was added. After mixing the ingredients, formulation flowability was determined on the basis of Carr’s index (21) and then formulations were compressed using a tablet press equipped with 14 mm flat punches. At the end, physicochemical tests including appearance, thickness, uniformity of weight, hardness, friability and disintegration time were conducted on tablets.

**Table 1:** The extra-granular components of series A, B, C, D, E and F ibuprofen ODT formulations

Extra-granular components							
**Ibuprofen** **(%)**	**Croscarmellose ** **(%)**	**Silicone dioxide** **(%)**	**Magnesium stearate ** **(%)**	***Aspartame*** ***(%)***	***Xylitol*** ***(%)***	Formulation	Series
**16.67**	**6.50**	**0.18**	**0.33**	**—**	**—**	F_1_	A
**16.67**	**6.50**	**0.18**	**1 .79**	**—**	**—**	F_2_	
**16.67**	**6.50**	**0.18**	**0.33**	**—**	**—**	F_3_	
**16.67**	**6.50**	**0.18**	**0.33**	**—**	**—**	F_4_	
**16.67**	**6.50**	**0.18**	**0.33**	**—**	**—**	F_5_	
**16.67**	**6.50**	**0.18**	**0.33**	**—**	**—**	F_6_	
**16.67**	**6.50**	**0.18**	**0.33**	**—**	**—**	F_7_	
**16.67**	**6.50**	**0.18**	**0.33**	**—**	**—**	F_8_	
**16.67**	**6.50**	**0.18**	**0.33**	**—**	**—**	F_9_	B
**16.67**	**6.50**	**0.18**	**0.33**	**—**	**—**	F_10_	
**16.67**	**10.00**	**0.18**	**0.33**	**—**	**—**	F_11_	
**16.67**	**15.00**	**0.18**	**0.33**	**—**	**—**	F_12_	
**16.67**	**20.00**	**0.18**	**0.33**	**—**	**—**	F_13_	
**16.67**	**20.51**	***―***	**―**	**—**	**—**	F_14_	
**16.67**	**―**	**―**	**―**	**―**	**―**	F_15_	C
**―**	**―**	**―**	**―**	**―**	**―**	F_16_	
**―**	**―**	**―**	**―**	**―**	**―**	F_17_	
**―**	**―**	**―**	**―**	**―**	**―**	F_18_	
**―**	**12.25**	**―**	**―**	**―**	**―**	F_19_	
**―**	**―**	**―**	**―**	**―**	**25.00 **	F_17a_	D
**―**	**―**	**―**	**―**	**25.00 **	**―**	F_17b_	
**―**	**―**	**―**	**―**	**―**	**―**	F_20_	E
**―**	**―**	**―**	**―**	**―**	**25.00 **	F_20a_	
**―**	**―**	**―**	**―**	**―**	**―**	F_21_	F
**―**	**―**	**―**	**―**	**―**	**50.00**	F_21a_	
**―**	**―**	**―**	**―**	**―**	**―**	F_22_	
**―**	**―**	**―**	**―**	**―**	**50.00**	F_22a_	

In series B formulations, polyethylene glycol 1000 was substituted by polyvinyl pyrrolidone in the intra-granular part and magnesium stearate and silicone dioxide were omitted from the extra-granular part of formulation. The granules of this series consisted of polyvinyl pyrrolidone and glucose. A 10% solution of polyvinyl pyrrolidone was prepared in alcohol for making these granules. Then, the stages used to prepare series A granules were repeated.

In series C formulations, ibuprofen, croscarmellose and glucose were used together in the granules. Series D formulations were made by the addition of xylitol and aspartame to formulation F_17_, extra-granularly. In series E formulations, alcohol was substituted by water in the polyvinyl pyrrolidone solution of formulation F_17_ and as a result formulation F_20_ was made. Then by adding xylitol to formulation F_20_, formulation F_20a_ was made. In series F formulations, alcohol was again used in the polyvinyl pyrrolidone solution and glucose was omitted from it. The method used for preparation of series C, D, E and F formulations and physicochemical tests conducted were the same as series B formulations.

**Table 2:** The intra-granular components of series A, B, C, D, E and F ibuprofen ODT formulations

Intra-granular components							
**Ibuprofen** **(%)**	**Croscarmellose ** **(%)**	**Polyvinyl pyrrolidone ** **(%)**	**Polyethylene glycol** **1000 (%) **	**Glucose** **(%) **	**Sucrose** **(%) **	Formulation	Series
**—**	**—**	**—**	**15.26**	**61.06**	**—**	F_1_	A
**—**	**—**	**—**	**14.97**	**59.89**	**—**	F_2_	
**—**	**—**	**—**	**12.72**	**63.60**	**—**	F_3_	
**—**	**—**	**—**	**10.90**	**65.42**	**—**	F_4_	
**—**	**—**	**—**	**19.08**	**57.24**	**—**	F_5_	
**—**	**—**	**—**	**25.44**	**50.88**	**—**	F_6_	
**—**	**—**	**—**	**15.26**	**—**	**61.06**	F_7_	
**—**	**—**	**—**	**19.08**	**—**	**57.24**	F_8_	B
**—**	**—**	**15.26**	**—**	**61.06**	**—**	F_9_	
**—**	**—**	**2.94**	**—**	**73.38**	**—**	F_10_	
**—**	**—**	**2.80**	**—**	**70.02**	**—**	F_11_	
**—**	**—**	**2.61**	**—**	**65.21**	**—**	F_12_	
**—**	**—**	**2.42**	**—**	**60.40**	**—**	F_13_	
**—**	**—**	**2.42**	**—**	**60.40**	**—**	F_14_	C
**―**	**20.51**	**2.42**	**―**	**60.40**	**―**	F_15_	
**16.67**	**20.51**	**2.42**	**―**	**60.40**	**―**	F_16_	
**16.67**	**24.51**	**2.42**	**―**	**56.40**	**―**	F_17_	
**16.67**	**28.51**	**2.42**	**―**	**52.40**	**―**	F_18_	
**16.67**	**12.255**	**2.42**	**―**	**56.40**	**―**	F_19_	
**12.50**	**18.38**	**1.82**	**―**	**42.30**	**―**	F_17a_	D
**12.50**	**18.38**	**1.82**	**―**	**42.30**	**―**	F_17b_	
**16.67**	**24.51**	**2.42**	**―**	**56.40**	**―**	F_20_	E
**12.50**	**18.38**	**1.82**	**―**	**42.30**	**―**	F_20a_	
**30.77**	**24.51**	**2.42**	**―**	**42.30**	**―**	F_21_	F
**15.385**	**12.255**	**1.21**	**―**	**21.15**	**―**	F_21a_	
**73.07**	**24.51**	**2.42**	**―**	**―**	**―**	F_22_	
**36.535**	**12.255**	**1.21**	**―**	**―**	**―**	F_22a_	

*Physicochemical tests conducted on ibuprofen ODT formulations*

The physicochemical characteristics of different ibuprofen ODT formulations prepared including power/granule flowability, appearance, thickness, uniformity of weight, hardness, friability and disintegration time were investigated. All these physicochemical tests were conducted in the standard way (5, 21, 24, 25), except for the disintegration time. An ODT formulation should disintegrate or dissolve in water quickly. Hence, for the purpose of conducting the disintegration time test, 6 tablets from each formulation were chosen randomly and each individually dropped into a basket connected to the wall of a beaker containing 10 mL pH 7.2 phosphate buffer (same as the saliva’s pH). Then, the beaker was placed on a shaker with a speed of 30 rpm, for imitation of lightly flow of saliva. In the next step, the duration of time required for disintegration of tablets were recorded. This test was performed at room temperature.

*Preparation of complementary formulations and taste masking*

Results of physicochemical tests conducted on formulation F_22a _(in series F) was found to be acceptable, making it the chosen formulation for further studies. Then, by adding various flavorants and sweeteners to this formulation, complementary series of formulations named G and H were prepared. At the next stage, these formulations were studied from the view point of user’s acceptance. Ten healthy and non-smoking volunteers; 5 females and 5 males, aged between 23 to 27 years; took part in this study and expressed their opinion on the appropriateness of the taste of formulated tablets. This was based on a scale from 1 to 5, with 1 representing the best formulation and 5 the worst formulation. It should be mentioned that the test formulations were coded in order to prevent any bias in the volunteers. Following the comparison of the taste of formulations, the most suitable formulation was considered as the ultimate formulation. This formulation was studied in terms of physicochemical tests conducted on the previous formulations, as well as the assay of active ingredient. HPLC was used for the assay of active ingredient after weighting and powdering 20 tablets randomly (26).

**Table 3:** Series G and H ibuprofen ODT formulations

Intra-granular components		Extra-granular components						Formulation	Series
**Intra-granular component of F**_22a_ ***(%)***	**Intra-granular component of F**_22a_ **withessence (%)**						**Saccharin(%)**		
		***Aspartame(%)***	***Xylitol(%)***		**Isomalt(%)**				
**―**	**66.67**	**―**	**33.33**		**―**		**―**	F_22b_	G
**―**	**50.00**	**―**	**50.00**		**―**		**―**	F_22c_	
**―**	**40.00**	**―**	**60.00**		**―**		**―**	F_22d_	
**―**	**33.33**	**―**	**66.67**		**―**		**―**	F_22e_	
**―**	**66.67**	**―**	**33.33**		**―**		**―**	F_22f_	
**―**	**50.00**	**―**	**50.00**		**―**		**―**	F_22g_	
**―**	**40.00**	**―**	**60.00**		**―**		**―**	F_22h_	
**―**	**33.33**	**―**	**66.67**		**―**		**―**	F_22i_	
**50.00**	**―**	**―**	**―**		**50.00**		**―**	F_22j_	H
**50.00**	**―**	**―**	**―**		**―**		**50.00**	F_22k_	
**66.66**	**―**	**16.67**	**16.67**		**―**		**―**	F_22l_	
**50.00**	**―**	**25.00**	**25.00**		**―**		**―**	F_22m_	
**66.66**	**―**	**―**	**16.67**		**16.67**		**―**	F_22n_	
**50.00**	**―**	**―**	**25.00**		**25.00**		**―**	F_22o_	
**66.66**	**―**	**―**	**16.67**		**―**		**16.67**	F_22p_	
**66.67**	**―**	**―**	**22.22**		**―**		**11.11**	F_22q_	
**66.67**	**―**	**―**	**11.11**		**―**		**22.22**	F_22r_	
**60.00**	**―**	**―**	**20.00**		**―**		**20.00**	F_22s_	

*Statistical analysis*

For the purpose of statistical comparison of the results obtained, student’s t-test was used in the cases where two samples were compared and the two-tailed ANOVA test in cases where over two samples were compared. In general, in cases where significant differences existed in the ANOVA test, Tukey post*-*hoc test was used to specify those samples having significant differences with each other. 

**Figure 1:**
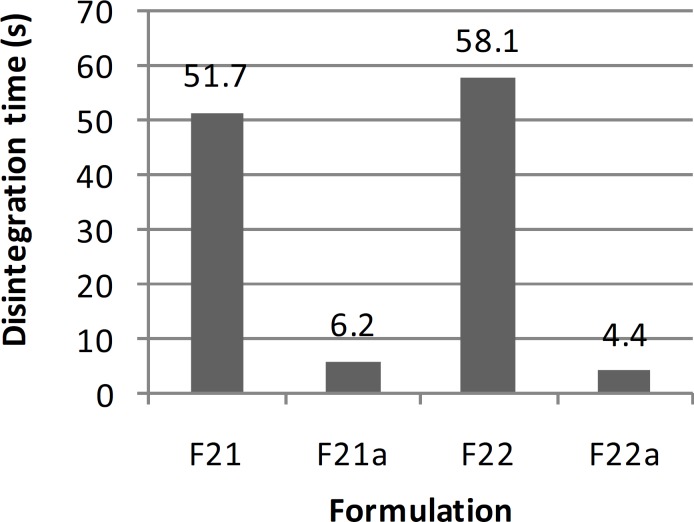
Mean disintegration time of series F ibuprofen ODT formulations (n = 6).

**Figure 2:**
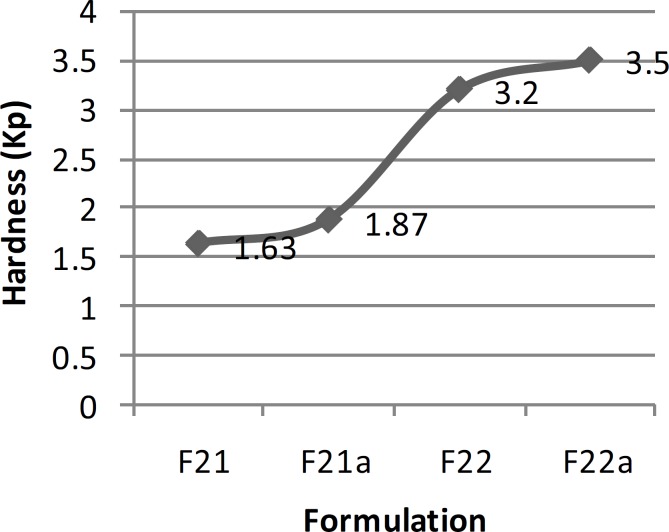
Mean hardness of series F ibuprofen ODT formulations (n = 10).

In order to study qualitative data (taste studies), Friedman and Wilcoxon paired statistical tests were used. The Friedman test shows the existence or non-existence of significant difference between formulations and Wilcoxon paired test compares formulations with each other pair-wise, in order to specify samples with significant differences. P-values less than 0.5 were considered as significant in all the above-mentioned tests. 

## Results and Discussion

Ibuprofen is an effective and widely used non-steroidal anti-inflammatory drug, with an extensive use in adults and children for relief of pain, fever and inflammation. The aim of this study was the preparation of a novel simple matrix-type ibuprofen ODT formulation, using special polymers, water-soluble excipients, super-disintegrants and quickly soluble granules. This formulation strategy could be cost-beneficial and can be easily adopted by the pharmaceutical companies. 

*Results of ibuprofen studies*

At first, physicochemical specifications of ibuprofen including organoleptic characteristics, flowability, compressibility, disintegration time of compacted powder and powder purity were investigated.

The results showed that ibuprofen is a white powder with a slight characteristic odor and undesirable taste, complying with the specifications (12, 13, 27). Hence, in order to improve its taste, the use of flavorants and sweeteners seems to be essential. The results obtained from flowability (poor flow), compressibility (n = 10, mean ± standard deviation of 0.30 KP ± 0.05 with the highest compression force) and disintegration time of compacted powder (over 30 min) indicated undesirable characteristics. Hence, for improving these characters, the use of appropriate ingredients and a suitable manufacturing method were necessary. The result of powder purity was 100.1% ± 0.2 (mean ± standard deviation; n = 3), showing compliance with the acceptable range of 98.5% to 101.0% mentioned in the literature (12).

*The results of ibuprofen ODT formulations*

Various formulations were prepared using different ingredients in eight series (A-H), and physicochemical properties of each series including flowability, appearance, thickness, uniformity of weight, hardness, friability and disintegration time were investigated. The results have been shown in Table 4. The flowability of series A formulations, except for F_5_ formulation, was worse than ibuprofen powder and there was a significant difference between the results obtained (ANOVA, p < 0.05). Formulation F_1_ was too sticky to be compressed and produced tablet. Also, formulations F_7_ and F_8_ were too sticky to measure their flowability and compressibility. Hence, series A formulations were rejected completely due to poor flowability, sticking to tablet press punches, undesirable appearance, unsuitable uniformity of weight, low hardness, high friability, and a long disintegration time (30 min).

**Table 4:** The results of physicochemical tests conducted on series A,B,C,D,E and F ibuprofen ODT formulations (results are presented as mean ± standard deviation).

(min or sec, n = 6)	Friability (%, n = 1)	Hardness (KP, n = 10)	Uniformity of weight (mg, n = 20)	Thickness (mm, n = 10)	Appearance (n = 10)	Flowability (n = 3)	**Formulation**
**―**	**―**	**―**	**―**	**―**	**―**	**Very poor**	F_1_
**15.1±1.8 sec**	**Not-acceptable**	**0.30±0.11**	**546.40±52.30**	**3.02±0.61**	**Undesirable**	**Very poor**	F_2_
**30.0±2.6 min**	**Not-acceptable**	**0.50±0.09**	**602.60±55.22**	**3.51±0.55**	**Undesirable**	**Very poor**	F_3_
**30.0±2.6 min**	**Not-acceptable**	**0.71±0.14**	**606.04±54.82**	**3.34±0.68**	**Undesirable**	**Between very poor and poor**	F_4_
**30.2±2.4 min**	**Not-acceptable**	**1.30±0.07**	**599.01±38.19**	**3.11±0.40**	**Undesirable**	**Moderate**	F_5_
**30.7±2.6 min**	**Not-acceptable**	**0.81±0.15**	**605.23±55.87**	**3.27±0.59**	**Undesirable**	**Poor**	F_6_
**―**	**―**	**―**	**―**	**―**	**―**	**―**	F_7_
**―**	**―**	**―**	**―**	**―**	**―**	**―**	F_8_
**―**	**―**	**―**	**―**	**―**	**―**	**―**	F_9_
**8.3±2.4 min**	**Not-acceptable**	**0.50±0.11**	**599.23±0.48**	**3.64±0.27**	**Desirable**	**Between good and excellent **	F_10_
**11.9±2.4 min**	**Not-acceptable**	**0.40±0.08**	**603.04±0.51**	**3.65±0.28**	**Desirable**	**Between good and excellent**	F_11_
**7.0±2.5 min**	**Not-acceptable**	**0.40±0.15**	**601.09±0.50**	**3.73±0.33**	**Desirable**	**Between good and excellent**	F_12_
**30.6±1.2 sec**	**Not-acceptable**	**0.40±0.20**	**602.65±0.64**	**3.83±0.37**	**Desirable**	**Between good and excellent**	F_13_
**4.8±1.5 sec**	**Not-acceptable**	**0.71±0.19**	**602.71±0.63**	**3.81±0.36**	**Desirable**	**Between good and excellent**	F_14_
**50.8±1.3 sec**	**Not-acceptable**	**0.45±0.19**	**598.77±0.22**	**3.72±0.39**	**Desirable**	**Excellent**	F_15_
**4.6±3.1 sec**	**Not-acceptable**	**0.81±0.11**	**602.14±0.09**	**3.71±0.28**	**Desirable**	**Excellent**	F_16_
**5.1±2.9 sec**	**Not-acceptable**	**2.03±0.08**	**601.29±0.02**	**3.83±0.21**	**Desirable**	**Excellent**	F_17_
**56.4±3.1 sec**	**Not-acceptable**	**0.95±0.13**	**602.65±0.07**	**3.80±0.32**	**Desirable**	**Excellent**	F_18_
**45.7±1.2 sec**	**Notacceptable**	**0.70±0.14**	**604.01±0.04**	**3.82±0.35**	**Undesirable**	**Excellent**	F_19_
**54.7±1.1 sec**	**Not-acceptable**	**1.22±0.15**	**779.01±0.18**	**4.48±0.25**	**Desirable**	**Between good and excellent**	F_17a_
**6.2±3.5 sec**	**Not-acceptable**	**0.71±0.11**	**781.60±0.11**	**4.52±0.28**	**Desirable**	**Excellent**	F_17b_
**6.1±2.7 sec**	**Not-acceptable**	**0.71±0.13**	**602.47±0.11**	**3.84±0.29**	**Desirable**	**Excellent**	F_20_
**49.8±1.3 sec**	**Not-acceptable**	**1.05±0.14**	**780.35±0.16**	**4.45±0.24**	**Desirable**	**Between good and excellent**	F_20a_
**51.7±1.1 sec**	**Not-acceptable**	**1.63±0.17**	**250.54±0.08**	**2.01±0.25**	**Desirable**	**Excellent**	F_21_
**6.2±3.0 sec**	**Not-acceptable**	**1.87±0.21**	**502.13±0.13**	**3.85±0.31**	**Desirable**	**Between good and excellent**	F_21a_
**58.1±0.9 sec**	**0.48**	**3.20±0.16**	**135.71±0.15**	**2.24±0.18**	**Desirable**	**Good**	F_22_
**4.4±2.7 sec**	**0.34**	**3.50±0.19**	**271.42±1.86**	**3.50±0.24**	**Desirable**	**Between poor and moderate **	F_22a_
**46.2±1.1 sec**	**0.45**	**3.24±0.17**	**225.02±0.15**	**3.10±0.25**	**Desirable**	**Between good and excellent**	F_22s_

**Figure 3:**
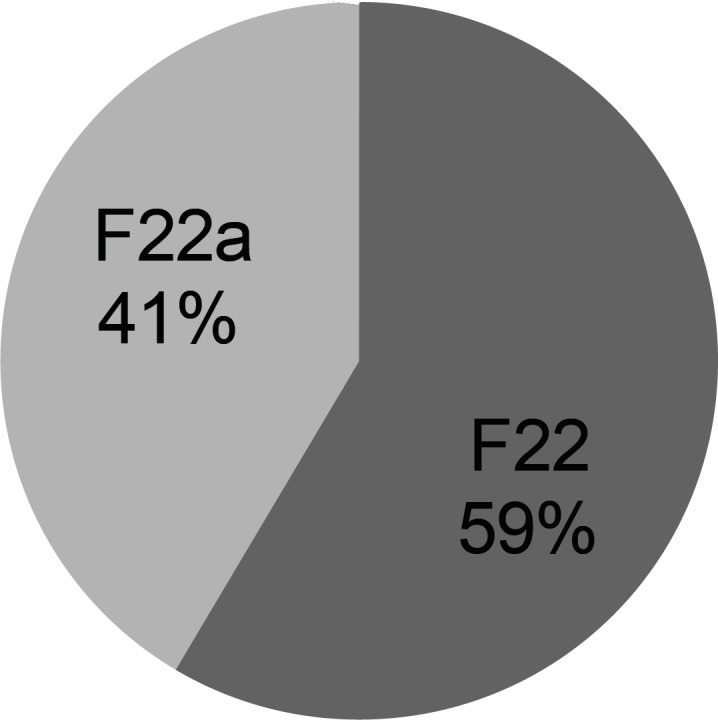
Friability (%) of series F ibuprofen ODT formulations (n = 1).

In series B formulations, polyethylene glycol 1000 was substituted by polyvinyl pyrrolidone in the intra-granular part of the formulation and magnesium stearate and silicon dioxide were omitted from the extra-granular part due to good flow and non-stickiness to punches and die. Except for formulation F_9_, which was too sticky for measuring flowability and compression, due to the low amount of glucose and high amount of polyvinyl pyrrolidone solution used, the other formulations could be evaluated. Statistical tests showed that there was no significant difference between the flowability of series B formulations (ANOVA, p > 0.05), but there were significant differences between the flowability of series B formulations with series A formulations and ibuprofen powder (ANOVA, p < 0.05). In addition, there was no significant difference between the results of thickness and uniformity of weight in series B formulations (ANOVA, p > 0.05). The major problems existing in series B formulations, resulting in their rejection and preparation of series C formulations were low hardness and high friability as well as inappropriate disintegration time in most cases (7-11 min).

Series C formulations, in which ibuprofen, croscarmellose and glucose were used intra-granularly; showed appropriate and acceptable results in terms of flowability, appearance of tablets (except for formulation F_19_), thickness, uniformity of weight and disintegration time. There was no significant difference between the results of thickness and uniformity of weight in series C formulations (ANOVA, p > 0.05). The results of disintegration time showed that, although there were significant differences between formulations F_16_ and F_17_ with the other series C formulations (ANOVA, Tukey post-hoc test, p < 0.05), never the less there was no significant difference between these two formulations (t-test, p > 0.05). The only problem was their low hardness and high friability. However, since ODT formulations should have a lower hardness in order to be disintegrated quickly within the buccal cavity, they would be expected to have a higher friability than conventional tablets and hence need special packaging. Therefore, formulation F_17_ was chosen as the selected ODT formulation among the formulations prepared, with a disintegration time of 5 sec.

**Figure 4:**
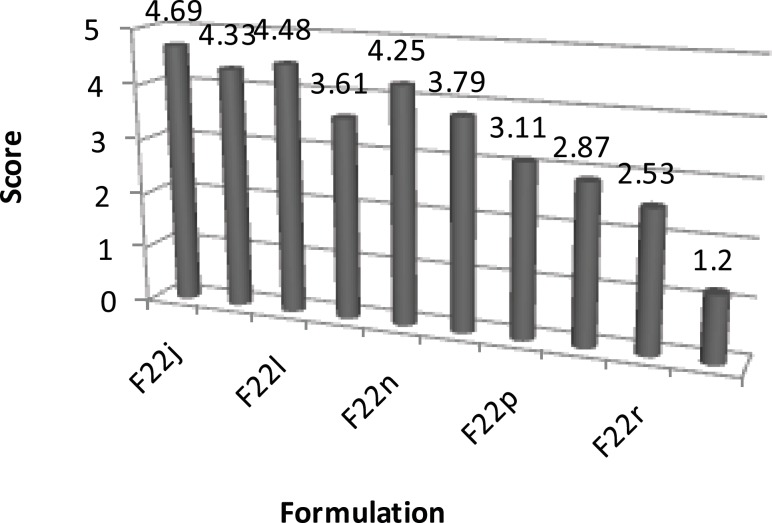
Taste of series H ibuprofen ODT formulations (scoring scale: the best = 1 and the worst = 5; n = 10).

In order to improve the taste of formulation F_17_, xylitol and aspartame were added extra-granularly and as a result series D formulations were made. In this series of formulations, the flowability of formulation F_17b_ was better than formulation F_17a_ and there was a significant difference between the results obtained (t-test, p < 0.05). In addition, there was a significant difference between the results of flowability, thickness, uniformity of weight and hardness of formulations F_17a _and F_17b_ with formulation F_17_ (ANOVA, Tukey post-hoc test, p < 0.05). Regarding the results obtained from the disintegration time, there was a significant difference between formulations F_17a _and F_17b_ (t-test, p < 0.05) and also formulations F_17a_ and F_17_ (t-test, p < 0.05). However, there was no significant difference between formulations F_17b_ and F_17_ (t-test, p > 0.05). Although, all the *in-vitro *results of formulations F_17a_ and F_17b_, except for hardness and friability, were within the acceptable but none of them had a desirable taste. Therefore, in series E formulations, in order to improve the taste, alcohol was substituted by water in the polyvinyl pyrrolidone solution. In these series of formulations, there was a significant difference between the results of flowability, thickness, uniformity of weight, hardness and disintegration time of formulations F_20 _and F_20a_ (t-test, p < 0.05) and a significant difference between the flowability results of formulations F_20_ and F_20a_ with formulation F_17_ (ANOVA, Tukey post-hoc test, p < 0.05). Regarding thickness, uniformity of weight and disintegration time, there was no significant difference between the results of formulations F_20_ and F_17_ (t-test, p > 0.05), but there was a significant difference between formulations F_20a_ and F_17_ (t-test, p < 0.05). There was also a significant difference between the hardness results of formulations F_20_ and F_20a_ with formulation F_17 _(ANOVA, Tukey post-hoc test, p < 0.05). Hardness of formulations F_20_ and F_20a_ was worse than F_17_. This could be due to the use of water instead of alcohol in the polyvinyl pyrrolidone solution. Moreover, series E formulations could not provide a better taste than the previous formulations studied. Hence, they were not found to be acceptable. In series F formulations, alcohol was used in the polyvinyl pyrrolidone solution, and glucose was omitted from the formulation. With respect to the results of flowability, thickness, uniformity of weight, hardness and disintegration time, there were significant differences between the results of series F formulations (ANOVA, p < 0.05). The results obtained from the physicochemical tests conducted on formulation F_22a_ (with a disintegration time of 4 sec), were desirable and acceptable in terms of all the tests conducted and as a result this formulation was chosen as the selected formulation. 

*Results obtained from complementary formulations and taste masking*

Series G and H complementary formulations were made by the addition of various flavorants and sweeteners to formulation F_22a_. After comparing their taste by considering the taste scores given by ten volunteers, formulation F_22s _was selected as the ultimate formulation. There was a significant difference between the taste results of formulation F_22a_ and series G and H formulations (Friedman test, p < 0.05). Moreover, there was a significant difference between the taste of formulation F_22s _with other formulations (Wilcoxon test, p < 0.05). Formulation F_22s_; which contained intra-granular ingredients of formulation F_22a_, including polyvinyl pyrrolidone, ibuprofen and croscarmellose and extra-granular components including xylitol and saccharin; was examined in terms of various physicochemical tests, as well as the assay of active ingredient. The results of physicochemical tests have been listed in Table 4. The assay of active ingredient (ibuprofen) of the final formulation (F_22s_), after conducting the related estimations, was determined to be 98.78% ± 0.05 (mean ± standard deviation; n = 3) which complied with the acceptable limit of 95.0-105.0% mentioned in the literature (26). Overall, all the physicochemical tests conducted on formulation F_22s _were found to be acceptable (particularly a disintegration time of 46 sec) and hence this formulation was chosen as the final formulation of this study.

**Table 5:** The results of taste studies (1 = best and 5 = worst) conducted on series F, G and H ibuprofen ODT formulations (n = 10; Mean ± SD).

**Series**	**Formulation**	**Taste score**
**F**	F_22a_	4.80 ± 0.32
**G**	F_22b_	4.92 ± 0.25
	F_22c_	4.76 ± 0.21
	F_22d_	4.68 ± 0.31
	F_22e_	4.37 ± 0.14
	F_22f_	4.89 ± 0.19
	F_22g_	4.78 ± 0.30
	F_22h_	4.53 ± 0.24
	F_22i_	4.45 ± 0.18
**H**	F_22j_	4.69 ± 0.26
	F_22k_	4.33 ± 0.17
	F_22l_	4.48 ± 0.13
	F_22m_	3.61 ± 0.39
	F_22n_	4.25 ± 0.27
	F_22o_	3.79 ± 0.32
	F_22p_	3.11 ± 0.79
	F_22q_	2.87 ± 0.74
	F_22r_	2.53 ± 0.79
	F_22s_	1.20 ± 0.42

## Conclusion

The aim of this study was to formulate a novel and simple matrix-type ibuprofen orally disintegrating tablet, capable of fast disintegration within the buccal cavity. Such a formulation could be found as a practical technology for adaptation in the pharmaceutical industry. The ultimate ibuprofen ODT formulation selected was formulation F_22s_, which contained polyvinyl pyrrolidone, ibuprofen and croscarmellose as the intra-granular components and xylitol along with saccharin as the extra-granular components. This formulation was examined in terms of various physicochemical tests and found to comply with all these tests, showing a disintegration time of 46 sec along with appropriate hardness and relatively low friability. This formulation also provided a desirable taste and hence could be considered as a promising ODT formulation. 

## References

[1] Remeth JD, Sfurti SS, Kailas KM (2009). Design and development of mucoadhesive acyclovir tablet. Iranian J. Pharm. Res.

[2] Ajit K, Manish B (2009). Development and evaluation of regioselective bilayer floating tablets of atenolol and lovastatin for biphasic release profile. Iranian J. Pharm. Res.

[3] Patel HR, Patel MM (2010). Development of osmotically controlled mucoadhesive Cup-Core (OCMC) tablet for the anti-inflammatory activity. Iranian J. Pharm. Res.

[4] Shafaati AR, Clark BJ (2003). Determination of aciclovir and its related substance guanine in bulk drug and tablet preparation by capillary electrophoresis. Iranian J. Pharm. Res.

[5] Allen LV, Popovich NG, Ansel HC (2005). Ansel’s Pharmaceutical Dosage Forms and Drug Delivery Systems.

[6] Bogner RH, Wilkosz MF (2004). Fast-Dissolving Tablets.

[7] Rangasamy M, Ayyasamy B, Raju S, Gummadevelly S (2009). Design and evaluation of the fast dissolving tablet of terbutaline sulfate. Asian J. Pharm.

[8] Remington TL (2004). Hand Book of Nonprescription Drugs.

[9] http://www.HughesMedicalCorp.com.

[10] Kearney P (2002). Modified Release Drug Delivery Technology.

[11] Zade PS, Kawtikwar PS, Sakarkar DM (2009). Formulation, evaluation and optimization of fast dissolving tablet containing tizanidine hydrochloride. Int. J. Pharm.Tech. Res.

[12] British Pharmacopoeia Commission Office (2005). British Pharmacopoeia (BP).

[13] Raffa RB (2005). Remington: The Science and Practice of Pharmacy.

[14] Sweetman SC (2007). Martindale: The Complete Drug Reference.

[15] Cada DJ (2004). Drug Facts and Comparisons. 8th ed. Facts and Comparisons®.

[16] Mizumoto T, Masuda Y, Yamamoto T, Yonemochi E, Terada K (2005). Formulation design of a novel fast disintegrating tablet. Int. J. Pharmaceutics.

[17] Suresh S, Pandit V, Joshi HP (2007). Preparation and evaluation of mouth dissolving tablets of salbutamol sulphate. Indian J. Pharm. Sci.

[18] Shukla D, Chakraborty S, Singh S, Mishra B (2009). Mouth dissolving tablets ΙΙ: An overview of evaluation techniques. Sci. Pharm. J.

[19] Sharma S, Gupta GD (2008). Formulation and characterization of fast-dissolving tablet of promethazine theoclate. Asian J. Pharm.

[20] Sharma S (2008). Orodispersable tablet. Online J. Pharm. Rev.

[21] Aulton ME (2002). Pharmaceutics: The Science Of Dosage Form Design.

[22] http://www.Ethypharm.com.

[23] Khawla A, Vincent L, Jee L, Graham P, Matthew S (2004). Fast-dissolving tablets.

[24] (2000). The United States Pharmacopoeia and National Formulary.

[25] British Pharmacopoeia Commission Office (2005). British Pharmacopoeia (BP).

[26] British Pharmacopoeia Commission Office (2005). British Pharmacopoeia (BP).

[27] The United States Pharmacopoeia and National Formulary (2006).

